# Analysis of factors influencing substance use craving among Chinese substance users

**DOI:** 10.3389/fpsyt.2022.1070215

**Published:** 2022-11-24

**Authors:** Huijuan Guo, Jizhi Wang, Siyuan Wang, Jiansong Zhou, Xiaoping Wang

**Affiliations:** ^1^Department of Psychiatry, National Clinical Research Center for Mental Disorders, The Second Xiangya Hospital of Central South University, Changsha, Hunan, China; ^2^Pingtang Compulsory Isolation Detoxification Institute in Hunan Province, Changsha, Hunan, China

**Keywords:** substance use, craving, influencing factor, substance use-related characteristics, psychological traits

## Abstract

**Background:**

Substance use has been a serious public safety issue. It not only affects the users’ physical and mental health but is also detrimental to social stability. To improve our understanding of this issue, the present study looked to examine the factors influencing substance use craving and develop interventions to reduce craving and relapse among substance users.

**Materials and methods:**

A total of 502 substance users were included in this study. Socio-demographic characteristics and substance use-related characteristics were recorded using self-developed forms. With regard to psychological traits, we used the self-esteem scale, the experience of shame scale, and the revised Cheek and Buss shyness scale to assess the self-esteem, shame, and shyness of substance users, respectively. The degree of substance use craving of substance users was assessed using the visual analog scale (VAS). Data were analyzed using independent samples *T*-test, Pearson correlation analysis, and multiple linear regression analysis, as appropriate.

**Results:**

The majority of the substance users were unmarried, employed, and with lower education levels. For substance use-related characteristics, the age of first use was 27.52 ± 8.30 years and the duration of substance use was 12.29 ± 7.72 years. The scores of their self-esteem, shame, and shyness were 25.65 ± 3.19, 57.26 ± 7.82, and 37.8 ± 7.13, respectively. All substance users rated the intensity of their substance use cravings using the VAS, which showed that the mean score was 2.83 ± 1.87. Multiple linear regression analysis showed that substance use craving was positively associated with the frequency of substance use (β = 0.186, *P* < 0.001), times of substance rehabilitation (β = 0.128, *P* = 0.003), shyness (β = 0.211, *P* < 0.001), and shame (β = 0.091, *P* = 0.033), and negatively associated with self-esteem (β = –0.117, *P* = 0.008).

**Conclusion:**

Factors and psychological traits related to substance use are important to account for substance use craving and relapse. Thus, our findings are helpful for a better understanding of the extent of substance use cravings among users and the selection of appropriate interventions to control the craving and relapse.

## Introduction

Substance use is a serious public safety problem, which not only harms users physically and psychologically (1, 2) but also is detrimental to social stability. For instance, substance users may get money for drugs illegally, such as through robbery, theft, and violent acts (3, 4). Therefore, the use and sale of substance have become a concern of the government and the whole of society. Despite significant efforts to reduce the prevalence and use of substance in some countries, the number of substance users is still increasing. According to the World Drug Report, approximately 200 million people used cannabis and 20 million people used cocaine in 2019, with the number of cannabis and cocaine users increasing by nearly 18 and 22%, respectively, over the past decade (5). Therefore, managing substance users is necessary for their drug withdrawal and preventing of future relapse.

To achieve the above goals, several studies have analyzed the factors affecting substance use and found that substance use craving plays an important role in post-cessation relapse and substance dependence (6–8). Craving is often defined as a strong, urgent desire for a particular substance. When a substance user attempts to change their current state of substance use, the level of craving may increase; if the craving is not satisfied, they may experience anxiety (9), depression (10), insomnia (11), and other psychological and somatic symptoms. Cravings also play a significant role in the diagnosis and treatment of substance use disorders. After being included in the Diagnostic and Statistical Manual of Mental Disorders-Fifth Edition (DSM-5), substance use craving has been considered a core component of addiction with important diagnostic implications (12), as well as one of the key goals of treatment for substance use disorders. Some drugs, such as methadone and buprenorphine, have been approved by the Food and Drug Administration (FDA) for the treatment of substance use disorders to reduce craving levels (13). Meanwhile, psychosocial interventions for addiction have also been developed for this phenomenon. For example, some cognitive behavioral therapies, including positive thinking interventions (14, 15) and behavioral therapy (16), have been used to help substance users control their cravings. However, despite the use of an increasing number of interventions, the cravings of substance users are still suboptimal. Some studies have shown that the relapse rate of substance use is well above 50% (17, 18). Therefore, research on the factors associated with substance use craving are warranted for developing more effective treatment strategies.

Previous studies exploring substance use craving-related factors suggested that substance use craving are positively associated with education degree (19), duration of abuse (20), and negatively associated with age (7). However, repeatability studies of the above results are still insufficient. Meanwhile, the sample sizes of the above studies were generally small, which might have affected the accuracy of the findings to some extent. Furthermore, in addition to assessing general demographic information and substance use-related characteristics, previous studies have also examined the role that psychological characteristics such as self-esteem, shame, or shyness play in substance users. However, they have typically focused on only one aspect of psychological traits. Therefore, given the above shortcomings of previous studies, the present study aimed to analyze factors related to substance use cravings with the combination of general demographic information, substance use-related characteristics and psychological traits.

## Materials and methods

### Participants

This cross-sectional study included all newly admitted substance users from two compulsory isolation centers in Hunan Province, China, from March to July 2019. The inclusion criteria were: (1) meeting the diagnostic criteria for substance-related and substance use disorders in the DSM-5; (2) able to understand the purpose of this study and sign the informed consent form. Subjects who met the DSM-5 diagnostic criteria for schizophrenia spectrum and other psychotic disorders, bipolar disorder and related disorders, depression, and other psychiatric disorders, as well as those who refused to participate in this study, were excluded. The study was approved by the Ethical Committees of the Second Xiangya Hospital of Central South University, the Lushan Compulsory Isolation and Rehabilitation Center, and the Baimalong Compulsory Isolation and Rehabilitation Center in Hunan Province, China.

### Instruments and evaluation

#### Socio-demographic characteristics

The general demographic information including gender, age, level of education, marital status, employment status, and residence was recorded for all the included participants. The level of education was divided into a low level (≤9 years, i.e., secondary school or below) and a high level of education (>9 years, i.e., high school and above).

#### Substance use-related characteristics

The participants were assessed on the categories of reasons, the answers were mainly divided into (1) blind curiosity, the pursuit of pleasure and excitement, and (2) other reasons. With regard to the frequency of substance use, a frequency of <4 times per week is defined as low, and ≥4 times per week is defined as high.

An open-ended question was used to obtain the types of drugs used by the subjects. All the drugs used by the subjects were categorized into new types of drugs (mainly referred to artificial chemical synthetic drugs that directly act on the central nervous system, including psychotropic drugs or drugs with both exciting and hallucinogenic effects, such as methamphetamine, ecstasy, and ketamine; these drugs are controlled by relevant laws and regulations in China) and traditional types of drugs (mainly referred to opium and heroin). The substance users were also divided into two categories based on the type of drugs they used: (1) users of traditional or new drugs and (2) users of mixed categories of drugs.

The times of drug rehabilitation (including the current admission) refer to the times of compulsory isolation and rehabilitation decided by the public security agency and executed by the judicial administrative agency. This variable was described using two categories: (1) once and (2) twice or more times.

The degree of substance use craving was assessed using the visual analog scale (VAS) (21), with 0 indicating no craving at all and 10 indicating extreme craving. VAS has been the most commonly used tool for the assessment of subjective craving with good reliability and validity (22).

#### Psychological traits

The subjects’ overall self-esteem was evaluated using the self-esteem scale (SES), a 10-item questionnaire compiled by Rosenberg in 1965 (23). It has shown good reliability and validity among Chinese populations (24). In the assessment, participants were asked to rate their attitudes against statements such as “Overall, I am very satisfied with myself” and “Overall, I think that I am a loser” on a 4-point scale (1 indicated “strongly agree,” 2 indicated “agree,” 3 indicated “disagree,” and 4 indicated “strongly disagree”); the total score of this scale was calculated by adding up the scores of all the items, with higher scores indicating higher degrees of self-esteem.

Experience of shame scale (ESS) was used to assess the shame experienced by the participants. The scale was jointly compiled and revised by Dr. Bernice Andrews from the Department of Psychology at Holloway College, University of London, and professor Qian Mingyi from the Department of Psychology, Peking University in 2002 (25). Each item was rated on a 4-point scale (1 indicated “not at all,” 2 indicated “occasional,” 3 indicated “sometimes,” and 4 indicated “often”), with the total score ranged 25–100. Higher scores indicated stronger feelings of shame.

Revised Cheek and Buss shyness scale (RCBS) was used to evaluate the shyness of the participants. The 13-item scale was originally compiled by Cheek and Buss, and the Chinese version was tested for reliability and validity by Xiang et al. (26), which showed strict equivalence across genders. In this scale, each item was rated on a 5-point scale, with 1 representing “strongly disagree” and 5 representing “strongly agree.” The total score ranged from 13 to 65, with higher scores indicating higher degrees of shyness.

### Statistical analysis

SPSS 23.0 software was used for statistical analysis. Continuous variables were presented as mean ± standard deviation, and categorical variables were presented as a number of cases and percentage. The degree of substance use craving was compared between categories using the independent samples *T*-test. The correlation between some variables and substance use craving was analyzed using Pearson correlation analysis. Multiple linear regression analysis was used to examine the factors influencing substance use craving. All the variables with a *p*-value of <0.05 in the *t*-test and Pearson correlation analysis were included as independent variables in the multiple linear regression model.

## Results

### Socio-demographic characteristics, substance use-related characteristics, and psychological traits of the substance users

A total of 534 new substance users were initially recruited, but 17 of them refused to participate in the study, and 15 were excluded due to the lack of data. Thus, a total of 502 participants (257 males and 245 females) were included in this study (Table 1). The participants were 20–65 years of age, with a mean age of 39.89 ± 8.87 years. Most of the substance users were single (72.1%), employed (72.9%), and had a lower level of education (80.3%). There was no significant difference in the number of substance users between genders (male vs. female) and between regions of residence (urban vs. rural).

**TABLE 1:** Socio-demographic characteristics, substance use-related characteristics, and psychological traits of the substance users.

Variables	Categories	Number/mean	Percentage/SD
**Socio-demographic characteristics**			
Age		39.89	8.87
Gender			
	Male	257	51.2
	Female	245	48.8
Marital status			
	Single	362	72.1
	Partnered	140	27.9
Employment			
	Unemployed	136	27.1
	Employed	366	72.9
Region of residence			
	Rural	256	51.0
	Urban	246	49.0
Level of education			
	Low	403	80.3
	High	99	19.7
**Substance use-related characteristics**			
Age of first substance use		27.52	8.30
Duration of substance use		12.29	7.72
Frequency of substance use (days per week)			
	Low	141	28.1
	High	361	71.9
Reasons for first substance use			
	Blind curiosity, the pursuit of pleasure and excitement	300	59.8
	Others	202	40.2
Types of substance used			
	New or traditional	413	82.3
	Mixed	89	17.7
Times of substance rehabilitation			
	Once	215	42.8
	Twice or more	287	57.2
**Psychological traits**			
Self-esteem		25.65	3.19
Shame		57.26	7.82
Shyness		37.85	7.13

This study found that the average age of first substance use was 27.52 ± 8.30 years and the average duration of substance use was 12.29 ± 7.72 years. The main reason for substance use was blind curiosity and the pursuit of pleasure and excitement (300 subjects, 59.8%), and most of them (361 subjects, 71.9%) used substance at a higher frequency (≥4 days/week), accounting for. By the time of this study, 42.8% of the substance users were admitted for compulsory isolated detoxification for the first time.

With regard to psychological features, we found that the average scores for self-esteem, shame, and shyness were 25.65 ± 3.19, 57.26 ± 7.82, and 37.85 ± 7.13, respectively.

All the participants rated the intensity of their substance use cravings using the VAS, which showed that the mean VAS score was 2.83 ± 1.87. The people who were single (*t* = –2.256, *P* = 0.025), had lower education degree (*t* = –2.787, *P* = 0.006), lower frequency of substance use (*t* = –6.254, *P* < 0.001), and higher time of drug rehabilitation (*t* = –4.314, *P* < 0.001) had higher substance use craving score (Table 2).

**TABLE 2:** Comparison of drug craving between categories of socio-demographic and substance use-related variables.

Variables	Categories	Drug craving score (Mean ± SD)	*t*	*P*
**Socio-demographic characteristics**				
Gender				
	Male	2.97 ± 1.88	1.696	0.091
	Female	2.69 ± 1.86		
Marital status				
	Single	2.95 ± 1.93	–2.256	0.025*
	Partnered	2.53 ± 1.68		
Employment				
	Unemployed	2.83 ± 1.87	–0.001	0.999
	Employed	2.83 ± 1.88		
Region of residence				
	Rural	2.89 ± 1.86	–0.731	0.465
	Urban	2.77 ± 1.89		
Level of education				
	Low	2.95 ± 1.91	–2.787	0.006**
	High	2.36 ± 1.63		
**Substance use-related characteristics**				
Frequency of substance use				
	Low	2.06 ± 1.65	–6.254	*P* < 0.001***
	High	3.13 ± 1.87		
Reasons for first substance use				
	Blind curiosity, the pursuit of pleasure and excitement	2.98 ± 1.88	–2.184	0.029*
	Others	2.61 ± 1.85		
Types of drugs used				
	New or traditional	2.79 ± 1.86	–1.065	0.287
	Mixed	3.02 ± 1.93		
Times of drug rehabilitation				
	Once	2.43 ± 1.62	–4.314	*P* < 0.001***
	Twice or more	3.13 ± 1.99		

Education: low level of education: ≤9 years of education, high level of education: >9 years of education. Frequency of substance use: low: <4 times per week, high: ≥4 times per week.

*P < 0.05, **P < 0.01, ***P < 0.001.

### Correlation of some continuous variables and substance use craving

This study found that the duration of substance use (*r* = 0.202, *P* < 0.001), shame (*r* = 0.185, *P* < 0.001), and shyness (*r* = 0.304, *P* < 0.001) were positively correlated with substance use cravings, while self-esteem (*r* = –0.224, *P* < 0.001) was negatively correlated with substance use cravings. The correlations between continuous variables and the substance use cravings are presented in Table 3.

**TABLE 3:** Correlation between drug craving and continuous socio-demographic, substance use-related, and psychological variables.

Variables	Pearson correlation	*P*-value
Age	0.063	0.157
Age of first substance use	–0.062	0.169
Duration of substance use	0.202	<0.001***
Shame	0.185	<0.001***
Shyness	0.304	<0.001***
Self-esteem	–0.224	<0.001***

***P < 0.001.

### Influencing factors of substance use craving

The multiple linear regression analysis suggested that substance use craving was positively associated with the frequency of substance use (β = 0.186, *P* < 0.001), times of substance rehabilitation (β = 0.128, *P* = 0.003), shyness (β = 0.211, *P* < 0.001) and shame (β = 0.091, *P* = 0.033), and negatively associated with self-esteem (β = –0.117, *P* = 0.008) (Table 4).

**TABLE 4:** Multiple linear regression analyses of drug craving.

Variables	*F*	*P*	*R* ^2^	Unstandardized coefficients	Standardized coefficients	*t*	*P*-value
	21.729	<0.001	0.180				
Constant				–0.561			
Shyness				0.055	0.211	4.918	<0.001***
Frequency of substance use				0.773	0.186	4.423	<0.001***
Times of drug rehabilitation				0.262	0.128	3.016	0.003**
Self-esteem				–0.069	–0.117	–2.680	0.008**
Shame				0.022	0.091	2.137	0.033*

*P < 0.05, **P < 0.01, ***P < 0.001.

## Discussion

In contrast to other studies, the present study assessed multiple aspects that might affect substance use cravings, i.e., socio-demographic characteristics, substance use-related characteristics, and psychological traits. Eventually, we found that the frequency of substance use, times of drug rehabilitation, shyness, and shame were positively correlated with substance use cravings, while self-esteem was negatively correlated with substance use craving.

With regard to substance use-related characteristics, the frequency of substance use and times of substance rehabilitation were found to be factors affecting substance use craving, with higher frequency of substance use and more times of substance rehabilitation associated with stronger craving. This is consistent with previous studies (27). As we have found that the frequency of substance use and times of detoxification are predictive of substance use craving, the management and intervention of substance users predicted to be at high risk should be the next step of our study.

Concerning psychological traits, shyness, self-esteem, and shame were found to be associated with substance use cravings. Shyness refers to an uncomfortable emotional and behavioral tendency to feel nervous, worried, or embarrassed due to excessive concern for oneself in interpersonal interactions and the fear of being judged by others. There are few studies on the correlation between substance use craving and shyness, while more studies have focused on the relationship between substance use and shyness, which showed opposite findings with the help of data from different populations. Studies involving high school students found that boys with a higher level of shyness used illicit substances more often than those with a lower level of shyness (28, 29), In contrast, a study on college students found that a lower level of shyness was associated with more severe substance use issues (30). A systematic review also showed that shyness was generally associated with less substance use (31). It is usually believed that the less shy a person is, the more curious they will be and the more likely they will try out drugs. In the present study, we found that higher levels of shyness were associated with higher levels of substance use craving, which might be explained by that psychoactive substances might have been used as a means to relieve social discomfort or inhibitions.

Shame refers to an overall negative feeling about some inappropriate behaviors or shortcomings of oneself. The present study indicated that the subjects’ shame was positively correlated with their substance use cravings. However, few studies have been conducted on the relationship between substance use craving and shame. A study found that the tendency of shame was generally positively associated with substance use problems among college undergraduates and jail inmates (32). This might be attributed to the interaction between substance abuse and shame, i.e., substance abuse might increase one’s feelings of shame, while the presence of shame might contribute to higher levels of substance use, which is regarded as an escape from their awkwardness. This interaction might lead to a vicious cycle, which needs to be verified in further studies.

Self-esteem is an emotional experience of self-love and the need to gain respect from others and the society based on self-evaluation. Structurally, it can be divided into global self-esteem and contingent self-esteem. Global self-esteem refers to a global judgment of one’s self-worth, while contingent self-esteem refers to the satisfaction people show when their performance meets a certain standard or fulfills certain interpersonal expectations. The present study found that lower self-esteem was associated with stronger substance use cravings, which is in line with a study by Kavas (33). Furthermore, some researchers have also found that contingent self-esteem might play a more important role in people’s behaviors. For example, Croker et al. found that people with lower global self-esteem but higher contingent self-esteem were more likely to use substances (34). Similarly, another study found that global self-esteem was negatively associated with substance use cravings while contingent self-esteem showed an opposite effect (35). A possible explanation is that contingent self-esteem is more unstable than global self-esteem, resulting in its greater impact on people’s substance use. For those with higher contingent self-esteem, the pursuit and maintenance of positive self-esteem often become the primary need that guides their thoughts, feelings, and behaviors; therefore, the dropping of contingent self-esteem might lead to higher proneness to low self-esteem state and negative emotions, which in turn contributes to substance abuse as an escape from negative emotions (36).

The present study showed no association between socio-demographic variables and substance use craving. However, several prior studies have found that some demographic variables are related to substance use craving, such as education and age. Generally, people with a higher level of education are more aware of the dangers of substance abuse, which might be why subjects with higher educational attainment only accounted for a small proportion in this study. However, a study found a significant positive relationship between the level of education and induced substance craving (19); according to their discussion, the autonomic nervous system becomes more active under persistent stress, especially educational stress, which makes people less resilient. Furthermore, increased cortisol levels may lead to higher substance use craving. In addition, age may be a key factor in substance use craving. Adolescence is a critical period for physical and mental development; as adolescents are still psychologically immature and curious about everything, they might be more vulnerable to substance use cravings. Evidence has shown that people who started using drugs during adolescence are more likely to become addicted, as compared with those who started using drugs later in life (37). However, the relationship between age and substance use was inconsistent across studies. For example, Viorel and Rudetsky et al. found that older adolescence was more likely to use illicit drugs (38, 39), while the study by Redland et al. showed that younger male adolescence (13–15 years old) were more likely to be involved in illicit substance use (40). This might be related to the different classifications of age groups in different studies. In any case, adolescents are in a critical period of physical and psychological development, and increased awareness of the danger of substance use is vitally important to prevent them from the harm of the drugs.

Despite the strengths of the present study, there are several limitations. First, this study is a cross-sectional study, which precluded us from following up the subjects for a longer period. Second, all the substance users included in this study were adults, and adolescents can be included in future studies for better investigation of the influence of age. Third, the factors examined in the present study are still limited, and more dimensions may be included in future studies.

## Conclusion

Substance use-related factors and psychological traits are also important factors affecting substance use craving. Our findings in the present study are helpful for a better understanding of substance use craving as well as the selection and development of appropriate intervention strategies to control cravings and reduce relapse.

## Data availability statement

The original contributions presented in this study are included in the article/supplementary material, further inquiries can be directed to the corresponding authors.

## Ethics statement

The studies involving human participants were reviewed and approved by the Ethical Committees of The Second Xiangya Hospital of Central South University. The patients/participants provided their written informed consent to participate in this study.

## Author contributions

HG conceived the study, performed data analyses, and drafted the manuscript. JW helped with data collection, performed data analysis, and drafted the manuscript. SW helped with data collection and performed data analysis. XW and JZ conceived, planned, and designed the study and revised the manuscript. All authors contributed to the article and approved the submitted version.
